# Mr. Shearly's Cases of Fungus Hæmatodes

**Published:** 1807-11

**Authors:** William Shearly

**Affiliations:** Naval Hospital, Deal


### Medical and Physical Journal.

VOL. XVIII.] NOVEMBER, 1807. [NO. 105.

*Printed for R. PHILLIPS, by W. Thorne, Red Lion Court, Fleet Street, London*

### *To the Editors of the Medical and Physical Journal.*

GENTLEMEN,

SITUATED in an extensive field of practical medicine, I have thought it a duty incumbent upon me to lay, from time to time, before the medical public, such select cases as appeared to be interesting and important. It is from such abodes of complicated disease and misery as Hospitals, that we may expect to derive important information, arising from experimental inquiry; experiments carried into execution after the usual methods have failed. When the new plan succeeds, and snatches from the grave the unhappy sufferer, that is the moment of triumph and exultation to the experimentalist. It unfortunately, however, happens frequently, that the person who makes known the result of such experiments is too sanguine in the cause, and by that very excess of anxiety to profit mankind, defeats his good intentions, by bestowing panegyrics on the recently discovered remedy, in general and indefinite terms, not considering sufficiently the idiosyncrasies of the person to whom it may have been administered.

*Naval Hospital, Deal,* I am, &c.

*Sept.* 28, 1807. WILLIAM SHEARLY.

### *Two Cases of Fungus Hæmatodes successfully treated by the external Use of Arsenic.*

CASE I. James Fraser was admitted into the hospital in the month of March 1804, labouring under the same disease which Mr. Hey describes in his Surgery, and to which he gives the appellation of Fungus Hæmatodes. This man received a blow upon the middle of the inner belly of the gastrocnemeus muscle of the right leg, which was followed by great pain. He went on with his duty for some time, without complaining to the surgeon of the ship. In a short time the skin cracked, and a substance resembling coagulated blood rose up. At the time of his admission, it was about 3 inches in length, 2½ in breadth, and one inch and ¼ elevated beyond the skin. It bled immediately upon applying the sponge to its surface, and that considerably. The patient's general health appeared to be fast declining. Solutions of the following substances, viz. Corrosive sublimate, zinc. vitriol, and lunar caustic, were tried without producing any advantage. A thought now struck us, that as the disease was produced in consequence of a blow (although not situated in a glandular part) it might possess something of a cancerous disposition, and as arsenic had been recommended for the cure of cancer, it was proposed to be tried externally after the following formula: ℞. Arsenic. Alb. in pulv. trit. ℈jj. Spt. Vin. Rect. ʒij. Aq. puræ ℥xjv. M. ft. Solutio. The parts were bathed with this night and morning, and dressed with dry lint, over which emollient dressings and a bandage were applied, the latter in such a manner as to make some considerable degree of pressure. The third day after this new method had been tried, a great difference for the better was observed in the fungus's aspect; and in the course of a fortnight this excrescence became level with the surrounding skin, and healthy granulations were perceived shooting over its whole extent. The solution was now omitted. Cicatrization went on rapidly, and the man was perfectly free from this disease in about a fortnight after the application of the arsenic was discontinued.

Case II. James M'Gregor, æt. 14, was admitted about two months after Frazer's dismissal. This boy was afflicted with the same disease (Fungus Hæmatodes) with these exceptions, that the fungus surrounded the patella, and the tumor did not rise so high as the man's did. This disease was treated in a similar manner as Case 1st, and in a short time the excrescences receded, and the lad was discharged perfectly well.

Observations.

Mr. Hey, in his valuable book, mentions the trial he made of several stimulating and caustic substances, which he found to be of no service in this dreadful malady (Fungus Hæmatodes). No internal medicine or external application appeared to arrest its progress, not even the dernier resort of surgery—the knife, as the stump put on the same appearance in a few days after amputation as the disease did prior to the opration. There can be no doubt that the fungus was organised from the tumour bleeding so readily on slightly applying a sponge to its surface. When the the disease has proceeded to great lengths, perhaps the internal as well as the external use of arsenic might prove highly advantageous. If the result of the foregoing cases should induce your readers to apply arsenic in this dreadful malady, the result of their experience I trust and hope will be made known.

### *Case of Ruptured Aorta near to its Origin.*

Thomas Jones, boatswain of his Majesty's ship Thames, early in the morning of the 24th of October, 1806, was heard to jump out of his hammock; a groan or two was also heard; and on a light being brought, he was found completely dead; his body was covered with perspiration. This man, from the account I received from Mr. James, the surgeon, had been doing his duty with his accustomed alacrity the day before, and appeared in a perfect state of health when he went to bed; indeed, he was never known to complain during the time he was on board the frigate, which was near a twelvemonth.

Dissection.

On opening the cavity of the thorax, and slitting open the pericardium, it was found distended with blood, and part of that in a coagulated state. On examining from whence this blood proceeded, the aorta was found ruptured, about an inch from the semilunar valves; this rupture was about half an inch in length. Small particles of calcareous matter were found deposited at the bottom of the three valves, and the corpora sesamoidea Auruntii, seemed to be composed in toto of this substance. The coats of the aorta were perfectly sound, as not the least deposition either of ossific or calcareous matter could be detected about the place where the rupture was situated. The abdominal viscera were perfectly healthy.

Remarks.

This man was very athletic, of a sanguineous temperament, had a short neck, and appeared in every respect a person much disposed to apoplexy. Might not spasm have been the cause of the rupture?

### *Case of Ruptured Liver in consequence of a Blow, unaccompanied with any external Marks of Violence.*

John Garrett, a seaman of his Majesty's ship Bellette, received a blow which was inflicted by the muzzle of a carronade, on the right hypochondrium. He received this blow immediately after taking a hearty meal. He fell down on the deck, complained of acute pain in the hypochondriac region, became shortly insensible, and expired about twenty minutes after he first received the blow.

### Dissection.

On an external inspection of the body, no marks of violence presented themselves; but on opening the abdomen, that cavity was distended with blood, and the liver was found ruptured. The rupture run through the right lobe, and extended to the lobulus spigelii, disuniting the gall-bladder partially from its relative situation.

### Remarks.

This case, as well as Blannan's,* points out the great necessity there exists in inspecting bodies who die suddenly, especially if there is any darkness hanging over the affair. I have often seen a coroner and jury perfectly satisfied with a superficial observance of a body under the above mentioned circumstances; and although hints have been thrown out as to the propriety of inspection, they have not been attended to. I am fully persuaded, numerous mysteries might be developed if inspections, under the above circumstances, were more generally adopted. The liver, in consequence of the distended state of the stomach, had the less power of resistance, and therefore received the whole of the shock which produced the rupture.

### *Case of Pleuritis accompanied with Carditis, the former terminating in Empyema, the latter in an extensive Deposition of coagulable Lymph, covering the whole Surface of the Heart, and likewise an Effusion of Water within the Cavity of the Pericardium.*

John Thompson, æt. 21, of his Majesty's ship Utrecht, of a fair complexion and irritable habit, was admitted into the hospital towards the beginning of the year 1804. Upon his admission he laboured under acute pleurisy, accompanied with decided symptoms of carditis. His pulse was 170, sometimes 180, and intermitted every 4th or 5th stroke. He was attacked with dreadful dyspnœa, attended with watchfulness and violent palpitations of the heart; he had no cessation from pain, referring it chiefly to the left side, and dare not lay in any other position than on his back. He once attempted to lay upon his left side, but the symptoms of suffocation were so severe that it deterred him from ever attempting the like again. To give some

* Vid. Blannan's case of ruptured colon, published in the Medical and Physical Journal, for November 1806.

idea idea what this poor fellow suffered from the violence of the pain, I really think that he did not sleep two hours together during the time he survived. Watchfulness prevailed to such an extent, that if the least noise was made, he would start up suddenly and appear much agitated. Every means was used to reduce and abate the violent symptoms, by abstracting great quantities of blood, joined with the application of perpetual blisters to the chest. Digitalis, opium, and chalybeates were prescribed, but every effort proved ineffectual to make the disease terminate favourably; and in about six weeks after his admission he terminated his existence, having been in a constant state of delirium three days previous to this event.

DISSECTION.

Upon opening the cavity of the thorax, a gallon * of pus was found to be contained in a cavity between the right lung and the ribs; the former had receded towards the left side, at least half a foot. The intercostal muscles of the right side were corroded, and the excavations filled up with layers of coagulable lymph. The pleura pulmonalis was so much thickened that it could very easily be divided into three distinct laminæ, each lamina being of considerable thickness. The pleura which lines the ribs was completely destroyed, and that part of the ribs which was originally invested by this membrane, was covered also with large clots of coagulable lymph, similar to the carnæa columnæ of the heart. The parenchymatous substance of the lungs appeared very healthy. The right lobe of the lungs was so much condensed, in consequence of the pressure made by the vast accumulation of matter, that it appeared about a quarter the size of the left. On opening the pericardium, it was found distended with water, and three pieces of coagulable lymph, as big as pigeons eggs, were found floating loose within. The surface of the heart was covered completely with this substance, (coagulable lymph.) The whole of the viscera contained in the abdomen were perfectly sound.

REMARKS.

It appears almost incredible, the patient could have survived the length of time he did under such a vast accumulation of disease, and the latter attacking parts so essential to life. The great anxiety, watchfulness, and intermission of the pulse is easily accounted for, by the appearances which

* We did not measure this fluid, but I think the quantity exceeded a gallon.

which presented themselves on dissection. This case differs widely from Poney's,* in regard to the diseased state of the heart, but is similar to it in one respect, viz. there being no organic affection of the lungs. The matter shewed no disposition to point, which rendered the subject so very obscure, that no operation could be performed; even had this been done, and the matter evacuated, the diseased state of the heart alone would have been sufficient to have caused his dissolution.

* Vid. a case of empyema successfully treated, Medical and Physical Journal, *January,* 1805.

